# Ship Typhus Fever in New Orleans

**Published:** 1848-04

**Authors:** 


*Ship Typhus Fever in New Orleans.*—The newspapers announce an extensive prevalence of this fever among the European immigrants of New Orleans. During the summer and early autumn of last year, the disease was by no means unknown in that city; but immigration was, of course, limited, for the St. Lawrence was then the channel, and Quebec, or, rather, its quarantine ground, thirty miles below, the point of debarcation. For half the year, *that* entrance is closed by ice; and during most of the other half, the climate of the Gulf of Mexico and the lower Mississippi, renders admittance into our country by *that* route extremely hazardous to foreigners on account of yellow fever. Thus, through the hot season, the chief current of immigration strikes our shores in the north—through the cold season, in the south. It has become perpetual, and in volume is on the increase. A vast majority of the immigrants, especially of those from Ireland, are miserably poor and dirty; they arrive with typhus, generated on the voyage, and they communicate it to those who receive and nurse them. It appears by the New Orleans Picayune of the 11th of February, that there were, at that time, more than five hundred cases of ship fever in the crowded wards of the Charity hospital; which thus seems likely to become as distinguished, in reference to typhus, as it has long been in reference to yellow fever. Two of the Sisters of Charity, who nobly devote themselves to the sick, had fallen victims, and one of the Professors of the Louisiana Medical School and several of its pupils, from labouring in the wards, had also been ill.

When and how will this alarming state of things come to an end? Is it not the duty of our Federal Government to interpose? We might insist, that the Governments of Europe shall so regulate the transportation of emigrants from their ports, as to prevent, if possible, the development of typhus among them. This, doubtless, could be accomplished by diminishing the number who are, at present, crowded on board each ship; secondly, by securing cleanliness and change of apparel and bedding; thirdly, by free ventilation; fourthly, by an abundant supply of healthy food; and lastly, by preventing the embarcation of any who are in the forming stage of the fever. Moreover, if there be any efficacy in Burnett's or Ledoyen's disinfecting compounds, emigrant ships are the places in which above all others they should be employed.

If the governments of the kingdoms of Europe would not, under due representations from our own, establish these or other sanitary regulations, the latter should prohibit the landing of all immigrants from ships which do not conform to them. As to the city of New Orleans, she ought, in immediate self-defence, to erect sheds upon the coast above or below the city, and not permit immigrants to enter it until they shall have passed several weeks in those temporary residences, under such hygienic discipline as her medical advisers might direct. The necessity for this will appear still more obvious on reading the following extract from the publication to which we have referred:

"The board of directors of the hospital have advices from Europe to the effect, that within the current year some forty thousand emigrants will sail for this place, if shipping can be had. Of these, it is judged, from the experience of the past year, that ten thousand will find their way to the hospital immediately upon landing. Some permanent arrangements should be made for this influx of disease; meanwhile, the present condition of the city requires immediate provision. The next arrival from Europe may overflow the hospital and infect the city."

One may at first be surprised that so great an immigration should take the circuitous New Orleans route, but we must remember that numerous ships come to that port for cotton, with little else than ballast, and that it is in them, at a low rate, that the poor emigrants come over.—*Western Journal of Medicine and Surgery.*

